# The Impact of Vitamin D Level on COVID-19 Infection: Systematic Review and Meta-Analysis

**DOI:** 10.3389/fpubh.2021.624559

**Published:** 2021-03-05

**Authors:** Amare Teshome, Aynishet Adane, Biruk Girma, Zeleke A. Mekonnen

**Affiliations:** ^1^Department of Dentistry, School of Medicine, College of Medicine, and Health Science, University of Gondar, Gondar, Ethiopia; ^2^Department of Internal Medicine, School of Medicine, College of Medicine and Health Science, University of Gondar, Gondar, Ethiopia; ^3^College of Medicine and Health Science, Institute of Public Health, University of Gondar, Gondar, Ethiopia

**Keywords:** vitamin D, COVID-19, SARS-CoV-2, review, meta-analysis

## Abstract

**Background:** Coronavirus disease (COVID-19) is a respiratory and systemic disorder caused by Severe Acute Respiratory Syndrome Coronavirus 2 (SARS-CoV-2) or novel Coronavirus (nCoV). To date, there is no proven curative treatment for this virus; as a result, prevention remains to be the best strategy to combat coronavirus infection (COVID-19). Vitamin D deficiency (VDD) has been proposed to play a role in coronavirus infection (COVID-19). However, there is no conclusive evidence on its impact on COVID-19 infection. Therefore, the present review aimed to summarize the available evidence regarding the association between Vitamin D levels and the risk of COVID-19 infection.

**Methods:** A systematic literature search of databases (PUBMED/MEDLINE, Cochrane/Wiley library, Scopus, and SciELO) were conducted from May 15, 2020, to December 20, 2020. Studies that assessed the effect of vitamin D level on COVID-19/SARS-2 infection were considered for the review. The qualities of the included studies were evaluated using the JBI tools. Meta-analysis with a random-effects model was conducted and odds ratio with their 95%CI were reported. This systematic review and meta-analysis are reported according to the preferred reporting items for systematic review and meta-analysis (PRISMA) guideline.

**Results:** The electronic and supplementary searches for this review yielded 318 records from which, only 14 of them met the inclusion criteria. The qualitative synthesis indicated that vitamin D deficient individuals were at higher risk of COVID-19 infection as compared to vitamin D sufficient patients. The pooled analysis showed that individuals with Vitamin-D deficiency were 80% more likely to acquire COVID-19 infection as compared to those who have sufficient Vitamin D levels (OR = 1.80; 95%CI: 1.72, 1.88). Begg's test also revealed that there was no significant publication bias between the studies (*P* = 0.764). The subgroup analysis revealed that the risk of acquiring COVID-19 infection was relatively higher in the case-control study design (OR = 1.81).

**Conclusions:** In conclusion, low serum 25 (OH) Vitamin-D level was significantly associated with a higher risk of COVID-19 infection. The limited currently available data suggest that sufficient Vitamin D level in serum is associated with a significantly decreased risk of COVID-19 infection.

## Introduction

Coronavirus disease (COVID-19) is a respiratory and systemic disorder caused by Severe Acute Respiratory Syndrome Coronavirus 2 (SARS-CoV-2) or novel Coronavirus (nCoV) (1). Severe Acute Respiratory Syndrome Coronavirus 2 is one of the coronavirus families, a family that was responsible for Middle East Respiratory Syndrome (MERS) and Severe Acute Respiratory Syndrome (SARS) (2). Coronavirus disease was first reported at Wuhan City, China in December 2020 (3, 4).

World Health Organization (WHO) declared the COVID-19 outbreak to be a public health emergency on January 30, 2020 (5) and a pandemic on March 11, 2020 (6). Till the 20th of December 2020, around 75 million COVID-19 cases and 1.7 million deaths were reported worldwide (7).

To date, there is no proven curative treatment for this virus; as a result, prevention remains to be the best strategy to combat COVID-19 pandemic. One of the preventive modalities is thought to be vitamin D (1, 25-dihydroxy vitamin D3) supplementation as evidenced by some observational studies. Some studies demonstrated that vitamin D deficiency (VDD) was associated with acute viral respiratory tract infection particularly caused by the influenza virus and acute lung injury (8, 9).

Vitamin D generally reduces the risk of microbial infection and death by modulating innate and adaptive immunity, and as a result of its antiviral and anti-inflammatory effects (10). Furthermore, vitamin D has a paramount effect on enhancing the expression of Angiotensin-converting enzyme 2(ACE-2), which is an important receptor mediating the pathogenesis of SARS-CoV-2 infection (11). Vitamin D can also enhance the expression of antioxidation-related genes, modulate adaptive immunity, and improves cellular immunity (12).

One challenge in halting this pandemic is the absence of proven treatment for COVID-19. Supplementation of vitamin D has been found to decrease viral acute respiratory infections, especially in persons with VDD (13). Considering the mechanisms of action of vitamin D, several studies have been conducted to evaluate the effect of vitamin D particularly in the context of the COVID−19 pandemic but continued to be an area of uncertainty and ongoing focus of attention (14, 15). However, the association between COVID-19 infection and VDD is still uncertain. Therefore, the present review is intended to summarize available literature regarding the impact of vitamin D level on COVID-19 infection.

## Methods and Materials

The results of this review are reported according to the Reporting Items for Systematic Reviews and Meta-Analyses (PRISMA) guideline (16), with the following research question: Is Vitamin-D deficiency associated with increased risk of COVID-19 infection?

### Inclusion and Exclusion Criteria

Using the PECO/PICO (population, exposure/Intervention, comparison/control, and Outcome) strategy, the studies that meet the following criteria were included in the study.

- **Populations:** Subjects participated in studies that assessed the impact of vitamin D level on COVID-19 infection.
- **Exposure/Intervention:** Vitamin D deficiency (VDD)
- **Comparison:** Sufficient vitamin D level
- **The outcome of the study:** COVID-19 infection (Positive or Negative).

### Exclusion Criteria

- Studies with no accessible full-text
- Ecological studies
- Studies that did not report specific outcomes quantitatively
- Abstracts, comments, reviews, posters, and editorial reviews.

### Information Sources and Search Strategy

We conducted a systematic search of databases (PubMed/Medline, Cochrane/Wiley library, Scopus, and SciELO) from May 15, 2020, to December 20, 2020, using key terms. Besides, reference lists of relevant studies were identified. The search strategy was built using a combination of keywords for the main axes of the research questions. The search strategy used terms related to (a) COVID-19/SARS-CoV-2 and (b) Vitamin D level/supplement. Search terms were pre-defined to allow a comprehensive search strategy that included text fields within records and Medical Subject Headings (MeSH terms) were used to help expand the search. We used Boolean operators (within each axis we combined keywords with the “OR” operator to expand the search and we then linked the search strategies for the two axes with the “AND” operator to narrow the search). No language restrictions were applied. When access to full-text articles was not available, authors were contacted through email.

### Study Selection

Database search results were combined and duplicate studies were removed using Endnote (version 7) and manually. After duplicates removed, titles and abstract screening was done and studies that were irrelevant to the overarching research question and outcome of interest were excluded. Full-text articles that warranted further investigation were assessed using the inclusion criteria. Two reviewers independently screened information at each stage. The disagreement was resolved by the involvement of a third independent reviewer.

### Data Extraction

Data extraction was done by two reviewers (AT, and ZA) using a standardized data extraction form. The following data were extracted from the included articles; characteristics of the study population, sample size, participant's status, and level of vitamin D, and outcomes of the study. The reported odds ratio (OR) or Risk ratio (RR) and the corresponding 95% CI or other relevant data were extracted. Any disagreement between the two reviewers was resolved by discussion and consensus. A third author (AA) was involved for persisted discrepancies. VDD and insufficiency was defined as a 25(OH) D level of <20 ng/mL (50 nmol/L) or as a 25(OH) D of 21–29 ng/ml (52.5–72.5 nmol/L), respectively, and sufficient/normal if 25(OH)D level was ≥30 ng/ml (17).

### Risk of Bias Assessment

The methodological quality of the studies was evaluated by two reviewers (AT and ZA) using JBI tools (18). The two authors' resolved disagreements in the assessment of the risk of bias by discussion and consensus, consulting a third author (BG) for any persistent disagreements. The kappa statistic was used to assess the level of agreement during the risk of bias assessment by the two authors.

### Study Outcomes

The primary outcome of the study was COVID-19 infection. We compared the risk of developing COVID-19 infection among VDD and normal Vitamin D levels.

### Statistical Analysis

Stata software (version 11.0, Stata Corporation, College Station, TX, US) was used to determine the pooled estimate. We used the Odds Ratio (RR) with a 95%CI to estimate the impact of Vitamin D status on COVID-19 infection. The heterogeneity was evaluated using the Cochran's *Q*-test, deriving its magnitude from the I square (*I*^2^) (19), and considered to have substantial heterogeneity if the *I*^2^ was >50%, and the random effect model is chosen; otherwise, the fixed-effect model is used. Furthermore, a sensitivity analysis was conducted by sequential removal of each study to evaluate each study's impact on the overall pooled effect. The publication bias was evaluated using Begg's tests. In all the analyses, a statistical assessment was two-tailed and considered statistically significant at a *p* < 0.05.

## Results

### Study Selection

**Figure 1:**
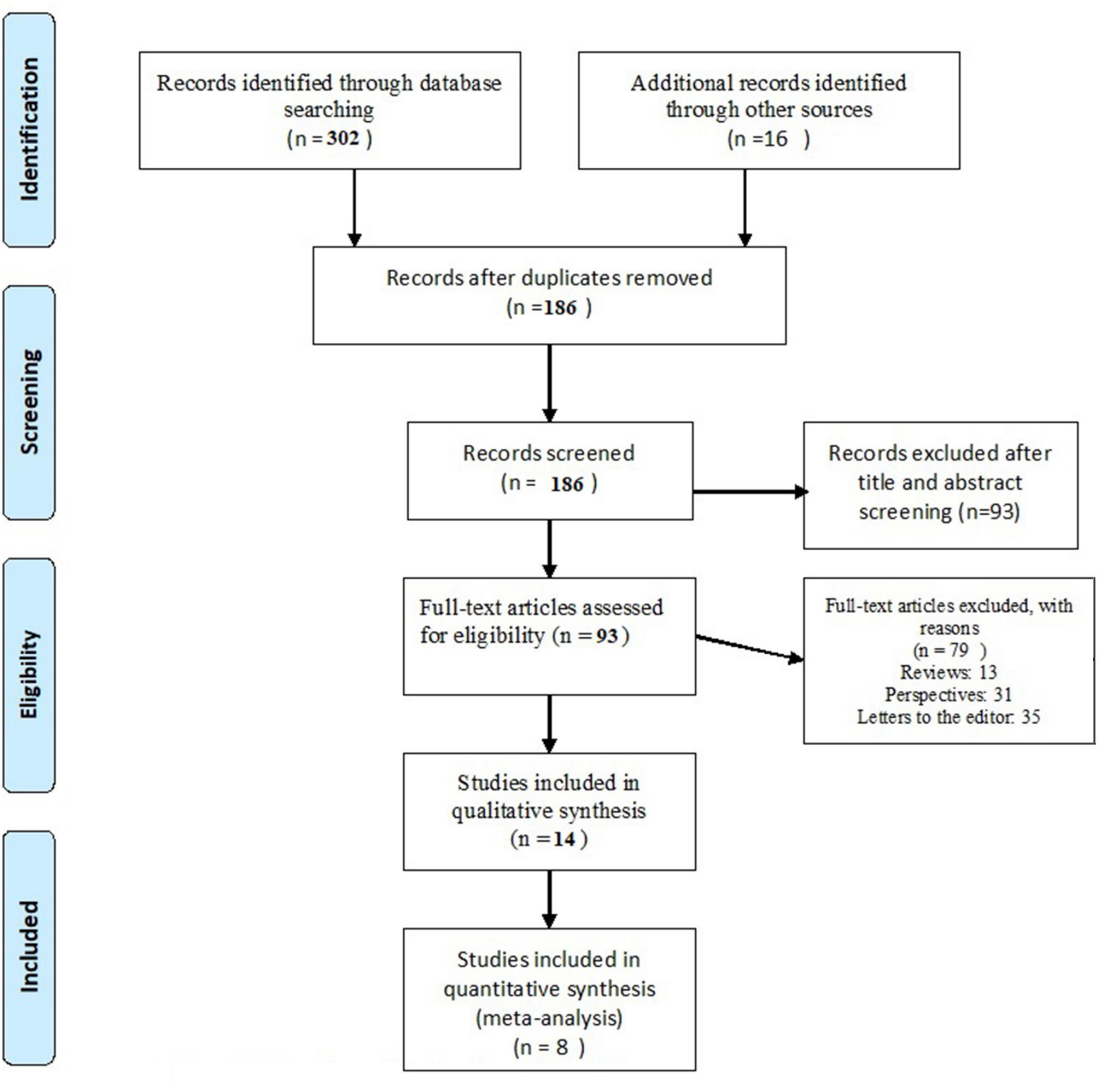
Flow diagram showing the article search process.

As shown in the flow diagram (Figure 1), 318 studies were searched from all databases. Of which, 132 were excluded as duplicates using Endnote 7 software and manually. The remaining 186 studies were filtered according to the titles and abstracts; 93 studies were excluded due to unrelated themes. A full-text review was done for the remaining 93 studies and identified 14 studies that meet the inclusion criteria for this review.

### Study Characteristics

Fourteen studies met the inclusion criteria with 91,120 participants. The sample size of the studies ranged from 134 to 79,381. The studies were conducted in Europe (14, 20–25), America (26, 27), and Asia (28–32). Moreover, the studies were cohort studies (14, 22, 25, 27, 30), case-control studies (20, 21, 26, 31, 32), cross-sectional studies (23, 28, 29) and interim audit (24) (see Table 1).

**Table 1:** Characteristics of included studies for the systematic review and meta-analysis.

**References**	**Country**	**Sample size**	**Study** **design**	**Participants**	**Outcome**	**Results and conclusion**				
1. Meltzer et al. (27)	USA	499	Cohort	VDD: 178 Vitamin-D sufficient: 321	COVID-19 infection	VDD (RR = 1.77) are at higher risk of Covid 19 infection as compared to vitamin D sufficient, with predicted COVID-19 rates in the vitamin D deficient group of 21.6%(95%CI [14.0–29.2%]) vs. 12.2%(95% CI [8.9–15.4%]) in the vitamin D sufficient group				
2. Raharusun et al. (30)	Indonesia	780	Cohort	COVID_19 patients	COVID-19 related mortality	The odds of death were higher in cases with insufficient Vitamin D status (OR = 7.63; *p* < 0.001) as compared to a normal level. When compared to cases with normal Vitamin D status, death was approximately 10.12 times more likely for Vitamin D deficient cases (OR = 10.12; *p* < 0.001)				
						**Vitamin D Covid-19 case (400) Death due to COVID-19 (380**)				
						Normal (= 388)	372 (93.0%)	16 (4.2%)		
						Insufficient (213)	26 (6.5%)	187 (49.1%)		
						VDD: (179)	2 (0.5%)	177 (46.7%)		
3. Merzon et al. (22)	Israel	7,807	Cohort	Covid-19 infected individuals	Vitamin D status among cases and Controls	Mean vitamin D level was significantly lower in COVID-19 patients than controls [19.00 ng/mL (95% CI: 18.41–19.59) vs. 20.55 (95% CI 20.32–20.78)]. Low plasma 25(OH) D level appears to be an independent risk factor for COVID-19 infection and hospitalization				
4. Hastie et al. (15, 25)	England	449	Cohort	COVID 19 patients	COVID-19 infection	There was no a potential link between vitamin D concentrations and risk of COVID-19 infection				
5. D'Avolio et al. (14)	Switzerland	187	cohort	SARS-CoV-2 PCR-positive	25-hydroxyvitamin D (25(OH) D) level among the cases and control group	Significantly lower 25(OH)D levels were found in COVID-19 patients (median value 11.1 ng/mL) patients compared with control groups (24.6 ng/mL). Vitamin D3 supplementation would be useful in the treatment of COVID-19 infection, in preventing more severe symptomatology and/or in reducing the presence of the virus in the upper respiratory tract and making the patients less infectious				
6. Abdollahi et al. (32)	Iran	402	Case-control	Covid-19 positive: 201 Covid-19 Negative: 201	Status of Vitamin D among the control and case groups	The level of serum 25(OH) vitamin D was significantly lower in COVID-19 positive patients (*p* = 0.02) and the results demonstrated that there was a significant relationship between the levels of serum 25(OH) vitamin D and the vulnerability to COVID-19				
							Cases		Controls	
							Insufficient 162 (80.5%)		132 (65.67%)	
							Sufficient 39 (19.4%)		69 (34.32%)	
						Vitamin D deficiency is one of the main predisposing factors associated with the vulnerability to coronavirus infection in the Iranian population				
7. Ye et al. (31)	China	142	Case-control	COVID-19 positive: 62 COVID-19 negative: 80	Status of vitamin D and severity of the diseases	Significantly higher rates of VDD were found in COVID-19 cases (41.9%) compared to control group (11.1%)				
							Cases	Control	Mild/mod	Severe
						Deficient	26 (42)	15 (19)	18 (36)	8 (80)
						Non-deficient	36 (58)	65 (81)	32 (64)	2 (20)
						VDD was a risk factor for COVID-19, especially for severe/critical cases				
8. Hernández et al. (20)	Spain	394	Case-control	Covid-19 positive: 197 Covid-19 Negative: 197	Vitamin D status and Covid-19 infection		Cases	Control		
						Deficient	82.2%	47.2%		
						Sufficient	17.8%	52.8%		
						Covid-19 positive patients had a lower vitamin D level than the control groups. Moreover, 25OHD levels are lower in hospitalized COVID-19 patients than controls. Serum 25OHD levels are significantly lower in hospitalized COVID-19 patients than in controls of similar age and sex, and that these differences remain significant even after adjusting for the main confounding factors. Patients with vitamin D supplements had an overall lower percentage of the combined severity endpoint and ICU admissions, as well as a shorter length of hospital stay, although these data did not reach statistical significance				
9. Kaufman et al. (26)	USA	79,381	Case-control	Covid-19 positive: 7,883 Covid-19negative: 71,498	Vitamin D status and Covid-19 infection	COVID-19	Positive	Negative		
						Deficient:	4,899	34,291		
						Adequate:	2,984	37,207		
						SARS-CoV-2 positivity is strongly and inversely associated with circulating 25(OH) D levels, a relationship that persists across latitudes, races/ethnicities, both sexes, and age ranges. Our findings provide impetus to explore the role of vitamin D supplementation in reducing the risk for SARS-CoV-2 infection and COVID-19 disease				
10. Yilmaz and Sen (21)	Turkey	85	Case-control	Covid-19 positive: 40 Covid-19 Negative: 45	Vitamin D status and Covid-19 infection		Cases	Control		
						Deficient:	29 (72.5%)	29		
						Normal:	11 (27.5%)	16		
						Patients with COVID-19 had significantly lower vitamin D levels 13.14 μg/L (4.19–69.28) than did the controls 34.81 (3.8–77.42) μg/L (p < .001)				
11. Maghbooli et al. (29)	Iran	235	Cross-sectional	COVID-19 Patients	COVID related morbidity and mortality	There was a significant association between vitamin D sufficiency and reduction in clinical severity, inpatient mortality, serum levels of C-reactive protein (CRP), and an increase in lymphocyte percentage. Only 9.7% of patients older than 40 years who were vitamin D sufficient succumbed to the infection compared to 20% who had a circulating level of 25(OH)D <30 ng/mL The severity of clinical outcomes from COVID-19 and mortality was dramatically reduced in patients who were vitamin D sufficient Improving vitamin D status in the general population and particularly hospitalized patients have a potential benefit in reducing the severity of morbidities and mortality associated with acquiring COVID-19				
									**25OHD** **≥30 (*****N*** **=** **77)**	**25OHD** **<** **30 (*****N*** **=** **158)**
								Inpatient mortality	9% (7)	20% (26)
								Severity-critical	63.6% (49)	77.2% (122)
12. De Smet et al. (23)	Belgium	186	Crossectional	SARS-CoV-2-infected patients	Analysis of 25(OH)D in COVID-19 patients	COVID-19 patients showed lower median 25(OH) D (18.6 ng/mL, IQR 12.6–25.3, vs. 21.5 ng/mL, IQR 13.9–65 30.8;) and higher VDD rates (58.6 vs. 45.2%)				
13. Panagiotou et al. (24)	England	134	Interim audit	Patients with COVID-19	Level of vitamin D among COVID-19 patients	A higher prevalence of VDD was observed in patients requiring intensive therapy unit compared to patients managed on medical wards. While mean serum 25(OH) D levels were comparable (*p* = 0.3), only 19% of ITU patients had 25(OH) D levels greater than 50 nmol/L vs. 39.1% of non-ITU patients (*p* = 0.02)				
14. Alguwaihes et al. (28)	Saudi Arabia	439	Crossectional study	COVID-19 patients	VDD and mortality	74.7% of COVID-19 patients had VDD, and patients with 25(OH) D < 12.5 nmol/l were 7 times at risk of mortality [AHR 7.0 (CI 1.7–28.2)]. VDD was significant predictors of mortality among hospitalized Covid-19 patients				

### Results of Individual Studies

Our synthesis indicated that being vitamin D deficient was at higher risk of COVID-19 infection as compared to vitamin D sufficient. This review has shown that when there is lower serum 25(OH) D level, the risk or susceptibility to COVID-19 increases (27, 29).

In a study conducted in England among hospitalized patients with COVID-19, VDD was associated with greater disease severity. The study indicated that a higher prevalence of Vitamin D deficiency (VDD) was observed in patients requiring intensive therapy unit (ITU) admission compared to patients managed on medical wards (24). A retrospective cohort study in Switzerland found significantly lower 25(OH)D levels in COVID-19 positive patients compared with negative patients (14). On contrary, findings from the UK biobank did not support the potential link between vitamin D level and risk of COVID-19 infection after adjusted for confounders (25).

A case-control study in Iran found that the level of serum 25(OH) vitamin D was significantly lower in COVID-19 positive patients (*p* = 0.02) and it demonstrated that there was a significant relationship between the levels of serum 25(OH) vitamin D and the vulnerability to COVID-19 (32). Ye et al. also revealed that VDD was a risk factor for COVID-19, especially for severe/critical cases (31). Moreover, other studies showed a lower vitamin D level in COVID-19 patients than the control group (20, 21, 26). A study done in Saudi Arabia found that 74.7% of COVID-19 patients had VDD and they were 7 times at risk of mortality [HR 7.0 (CI 1.7–28.2); *p* = 0.007] (28).

A study from Belgium revealed that VDD is a prevalent risk factor for severe COVID-19 infection (23). Maghbooli et al. indicated that 25(OH)D levels of ≥30 ng/mL were associated with a significant decrease in the severity of clinical outcomes related to a COVID-19 infection (29). A population-based study from Israeli also reported that low plasma 25(OH)D level appears to be an independent risk factor for COVID-19 infection and hospitalization (22). Also, it was indicated that the odds of death were higher in COVID-19 cases with insufficient vitamin D status (28, 30).

A study done in Iran found that Improving vitamin D status in the general population and particularly hospitalized patients have a potential benefit in reducing the severity of morbidities and mortality associated with acquiring COVID-19 (29). Moreover, D'Avolio et al. in Switzerland stated that Vitamin D3 supplementation would be useful in the treatment of COVID-19 infection, preventing more severe symptoms and/or in reducing the presence of the virus in the upper respiratory tract and making the patients less infectious (14) (Table 1).

### Risk of Bias Within Studies

The qualities of the included studies were evaluated based on the JBI critical appraisal checklist and studies with a quality assessment score of 50% and above were included in the review.

### Results of the Meta-Analysis

**Figure 2:**
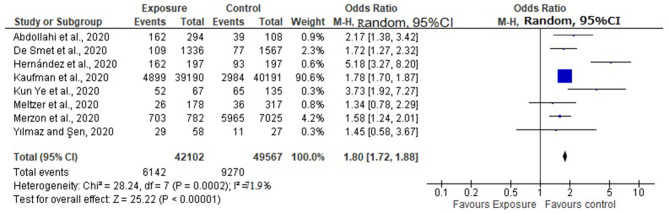
Pooled effect of Vitamin D status and COVID-19 infection.

**Figure 3:**
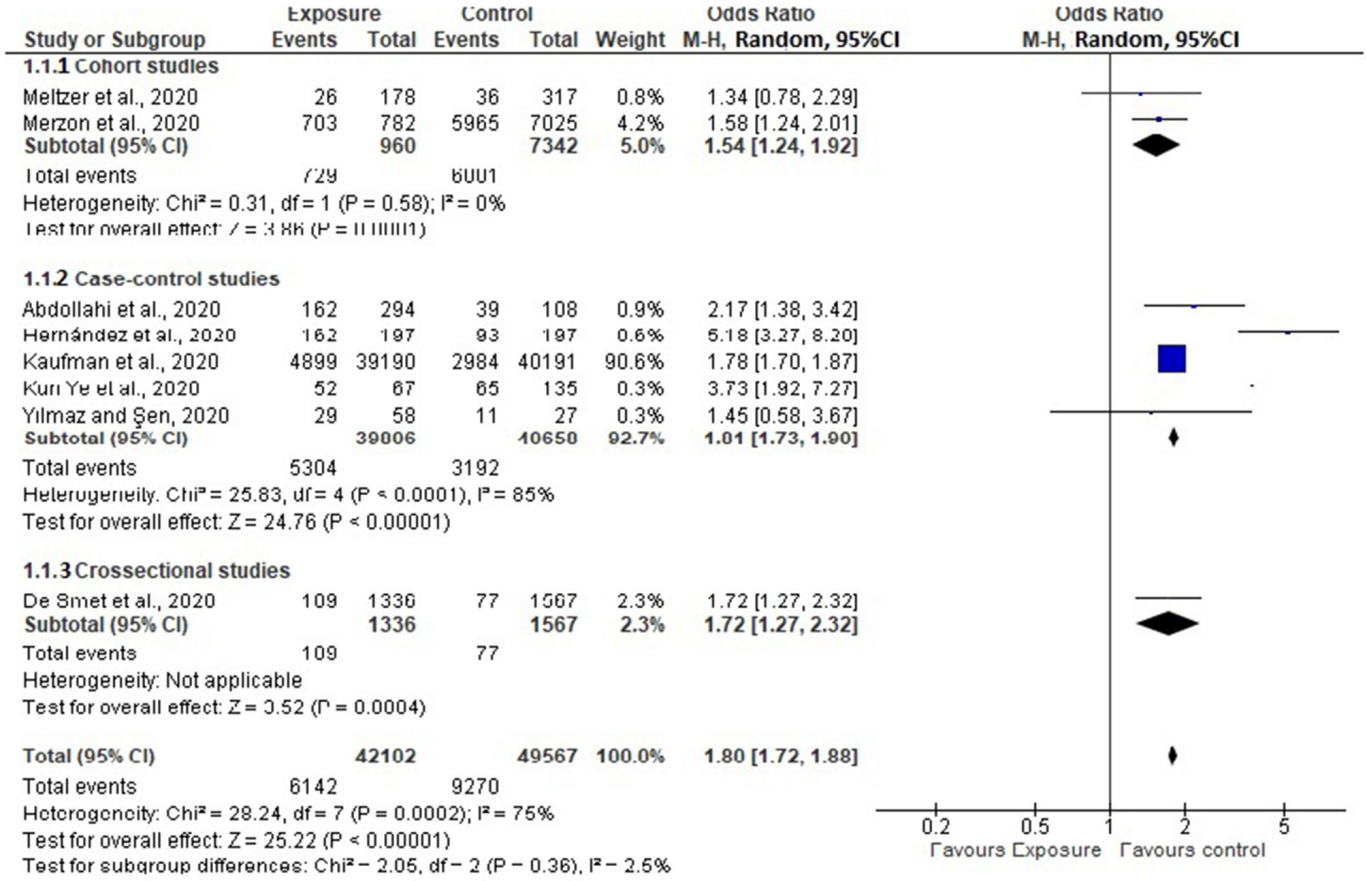
Subgroup analysis based on study design.

Eight of the 14 selected studies reported the impact of vitamin D level on COVID-19 infection (20–23, 26, 27, 31, 32). Overall, pooled OR in the random-effect model showed that VDD was associated with an increased risk of COVID-19 infection (OR = 1.80, 95% CI: 1.72, 1.88). Accordingly, those individuals with an insufficient level of Vitamin D are 80% more likely to acquire COVID-19 infection as compared to those who have a normal level of Vitamin D. The forest plot showed substantial heterogeneity with *I*^2^ of 79.1% (Figure 2). Begg's test revealed there was no significant publication bias between the studies (*P* = 0.764). Subgroup analysis revealed that the pooled effect of VDD was 1.81 in case-controlled studies (OR = 1.81, 95% CI: 173, 190) (Figure 3).

### Sensitivity Analysis

**Figure 4:**
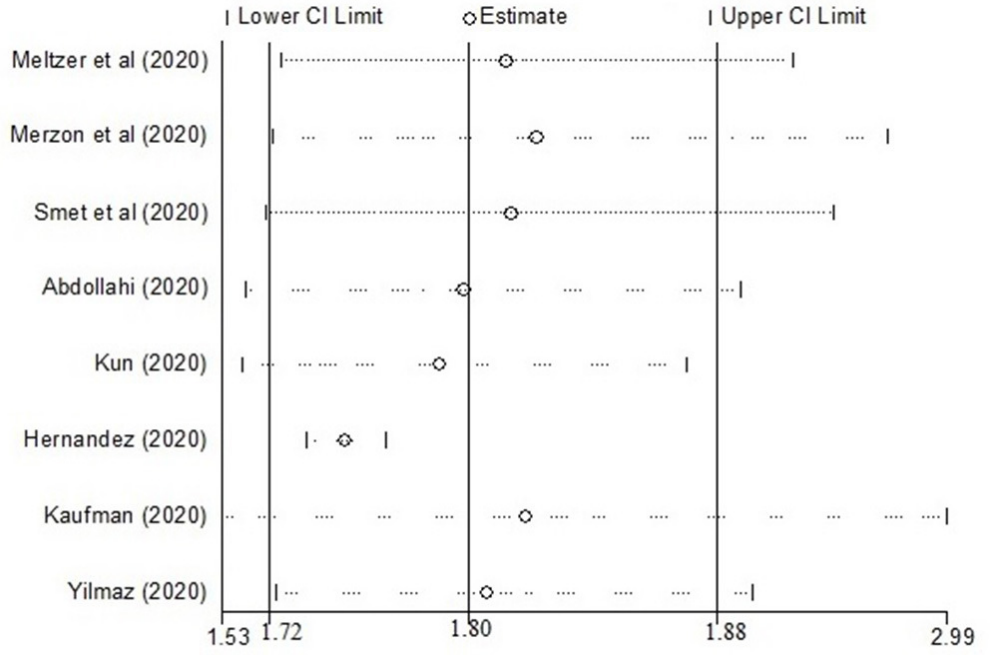
Sensitivity analysis showing the influential studies on the overall pooled effect.

The sensitivity analysis revealed that the studies done by Kaufman et al. (26) and Hernandez et al. (20) were the influential studies on the overall pooled effect (Figure 4).

## Discussion

In the present review, we observed a significant association between a low level of Vitamin D and the risk of acquiring COVID infection, which is supported by previous studies that revealed vitamin D has protective effects against acute respiratory infections (13). Moreover, a meta-analysis of randomized controlled trials showed that improving vitamin D status has been associated with a reduced risk of upper or lower respiratory tract infections (13). The possible role of vitamin D in infectious diseases like COVID-19 is explained by its regulatory role on acquired and innate immunity (33). Evidence also indicated that vitamin D might help in the treatment of COVID-19 by preventing the cytokine storm and subsequent ARDS which is commonly the cause of mortality (29, 34).

The pooled estimate showed that subjects with VDD were 80 % more likely to acquire COVID-19 infection (OR = 1.80; 95% CI: 1.72, 1.88), which is in line with the previous meta-analysis where vitamin D deficiency or insufficiency participants were at increased risk of COVID-19 infection (OR = 1.43, 95% CI: 1.00–2.05) (35). Besides, Ilie et al. reported that vitamin D levels are severely low in COVID-19 positive individuals and found a negative correlation between levels of mean vitamin D and COVID-19 infection (11).

This review showed that improving vitamin D status in the general population has a potential benefit in reducing the risk of acquiring COVID-19 infection. Evidence by Chandran et al. also recommends supplementation of vitamin D in patients with COVID-19 (36). A meta-analysis of randomized controlled trials (RCTs) concluded that the use of vitamin D supplements was associated with lower mortality in adults (37). A systematic review and meta-analysis on the effect of Vitamin D on ARTI reported that there is an inverse non-linear association between 25(OH)D concentration and risk of ARTIs (38). The evidence presented in this review showed promise for the use of Vitamin D supplementation to reduce the risk and severity of COVDI-19 infection.

### Limitation of the Study

Our study has some strengths and limitations. The main strength of the current review lies in our adherence to international standardized guidelines on the conduct and reporting of systematic reviews. We included studies only from peer-reviewed journals, which may have restricted our findings. However, some of the limitations of our study include; most of the included studies were hospital-based studies and the data were from secondary sources that become more prone to high risk of bias.

## Conclusion

In conclusion, low serum 25 (OH) Vitamin-D level was significantly associated with a higher risk of COVID-19 infection. The limited currently available data suggest that sufficient Vitamin D level in serum is associated with a significantly decreased risk of COVID-19 infection. Besides, further rigorous studies are needed to strengthen the evidence.

## Data Availability Statement

The original contributions presented in the study are included in the article/supplementary material, further inquiries can be directed to the corresponding author/s.

## Author Contributions

AT, BG, and AA did the article searching. ZM and AT performed the critical appraisal and data extraction. All authors conceived and designed this review, involved in data analysis, interpretation of results, write up of the manuscript, read, and approved the manuscript.

## Conflict of Interest

The authors declare that the research was conducted in the absence of any commercial or financial relationships that could be construed as a potential conflict of interest.

## Abbreviations

ACE: Angiotensin-Converting Enzyme
ARTI: Acute Respiratory Tract Infection
CDC: Centre for disease control and prevention
COVID-19: Corona Virus Disease 2019
HR: Hazard Regression
JBI: Johanna Briggs Institute
PECO: population, exposure, control, and outcomes
PICO: population, intervention, control, and outcomes
RCT: Randomized Controlled Trial
SARS: Severe acute respiratory syndrome
VDD: Vitamin D Deficiency.
